# Ultrasound-Assisted Extraction of Carnosic Acid and Rosmarinic Acid Using Ionic Liquid Solution from *Rosmarinus officinalis*

**DOI:** 10.3390/ijms130911027

**Published:** 2012-09-05

**Authors:** Ge Zu, Rongrui Zhang, Lei Yang, Chunhui Ma, Yuangang Zu, Wenjie Wang, Chunjian Zhao

**Affiliations:** State Engineering Laboratory for Bioresource Eco-Utilization, Northeast Forestry University, Harbin 150040, China; E-Mails: zygenefu@163.com (G.Z.); zhangrongrui1986@126.com (R.Z.); mchmchmchmch@163.com (C.M.); wjwang225@hotmail.com (W.W.); zcjsj@163.com (C.Z.)

**Keywords:** *Rosmarinus officinalis*, carnosic acid, rosmarinic acid, ionic liquid, ultrasound-assisted extraction, response surface methodology

## Abstract

Ionic liquid based, ultrasound-assisted extraction was successfully applied to the extraction of phenolcarboxylic acids, carnosic acid and rosmarinic acid, from *Rosmarinus officinalis*. Eight ionic liquids, with different cations and anions, were investigated in this work and [C_8_mim]Br was selected as the optimal solvent. Ultrasound extraction parameters, including soaking time, solid–liquid ratio, ultrasound power and time, and the number of extraction cycles, were discussed by single factor experiments and the main influence factors were optimized by response surface methodology. The proposed approach was demonstrated as having higher efficiency, shorter extraction time and as a new alternative for the extraction of carnosic acid and rosmarinic acid from *R. officinalis* compared with traditional reference extraction methods. Ionic liquids are considered to be green solvents, in the ultrasound-assisted extraction of key chemicals from medicinal plants, and show great potential.

## 1. Introduction

*Rosmarinus officinalis* (Lamiaceae) is a perennial herb with fragrant needle-like leaves. It is native to the Mediterranean region and has been cultivated in many other regions. The fresh and dried leaves of *R. officinalis* are widely used as flavors in different food products due to the powerful aromatic odor. Their essential oils are widely applied in the cosmetic industry producing various Cologne waters, bathing essences, hair lotions, shampoos and as a component of disinfectants and insecticides. Besides the aromatherapy application, carnosic acid and rosmarinic acid are the major ingredients of solvent extracts from *R. officinalis*. Carnosic acid is a naturally occurring phenolic diterpenoid, and many bioactivities of carnosic acid have been reported, antioxidant [1–4], antimicrobial [5], anti-inflammatory [6], anti-cancer [7], chemoprotective [8], neuroprotective [9], antiobesity [10] and so on. Rosmarinic acid has a number of biological activities such as antioxidant [11,12], anti-inflammatory [13], antimutagenicity [14], antiangiogenic [15], anti-apoptotic [16], antifibrotic [17], chemoprotective [18], neuroprotective [19], reduction of atopic dermatitis [20], photoprotection of keratinocytes [21] and prevention of Alzheimer’s disease [22].

In conventional methods, heat reflux extraction (HRE), maceration extraction (ME), stirring extraction (SE) and Soxhlet extraction (SLE), were the benchmark extraction techniques of carnosic and rosmarinic acid. But there are several disadvantages of these methods, such as the use of volatile and hazardous solvents, the long extraction time and more recovery energy.

To overcome the above-mentioned problems, environment friendly techniques become attractive following the development of “Green Chemistry”. Much wider attention has been given to applications of ultrasound-assisted extraction (UAE) [23] and microwave-assisted extraction (MAE) [24,25]. Among the two methods, UAE can more easily be scaled up for commercial production [23]. Also the UAE is a promising extraction technique that can offer high reproducibility in a shorter time, simplified manipulation, reduced solvent consumption and temperature and lower energy input, which has been widely used to extract analytes from many matrixes [26,27]. Ultrasound enhancement of extraction is attributed to the disruption of cell walls, particle-size reduction and the enhancement of the mass transfer of the cell content to the solvent caused by the collapse of the bubbles produced by cavitations [28,29]. The UAE is expeditious, inexpensive, efficient and an environmental protection alternative to conventional extraction techniques, which is also a well-established method in the processing of plant material, and in the extraction of analytes from different parts of plants.

Traditional extraction employs an organic solvent and/or an aqueous solution as the solution. The organic solvents used are generally toxic, flammable and volatile. Ionic liquid is a new green chemical revolution which excited the academia and chemical industries [30]. They normally consist of an organic cation, the most commonly used being dialkylimidazolium, *N*-alkylpyridinium and tetra-alkyl ammomium salts, and an inorganic anion such as Cl^−^, Br^−^, [HSO_4_]^−^, [BF_4_]^−^, [PF_6_]^−^. Ionic liquids are suitable and favorable novel solvents that could replace traditional volatile organic compounds as a result of their unique properties, such as the negligible vapor pressure, nonflammability, and the good solubility with organic and inorganic compounds [31]. Ionic liquids as solvent is of promising potential in the application of the preparation of various useful substances from plant samples such as alkaloids [23,24,32,33], stilbene [34], proanthocyanidins [35], quinines [36], lignans [37,38], coumarins [39] and free fatty acids [40].

The aim of this paper is to develop a rapid, effective, validated and environmentally friendly ionic liquid-based ultrasound-assisted extraction (ILUAE) approach for the extraction of carnosic acid and rosmarinic acid from this plant, comparing the results with the conventional reference methods. Herein we systematically investigated the performance of various ionic liquids with different cations and anions as well as the influential parameters of the UAE procedure. After the optimization of extraction conditions, the proposed extraction proved to be an efficient and excellent extraction of carnosic acid and rosmarinic acid from *R. officinalis.*

## 2. Results and Discussion

### 2.1. Screening of the Ionic Liquid-Based Extracting Solvent

The structure of ionic liquids has significant influence on their physicochemical properties, and would therefore be expected to affect the extraction efficiency of the target compounds [34,36], the effects of changing the anion and the alkyl chain length of the cation of 1-alkyl-3-methylimidazolium-type ionic liquids on the extraction efficiency were studied and the general trends observed are described below.

The anion identity is considered to be the most important and has the most dramatic effect on the properties [37]. Therefore, *N*-methylimidazolium based ionic liquids with five different anions (Cl^−^, Br^−^, NO_3_^−^, BF_4_^−^, OH^−^) were studied. All of the ionic liquids tested were sufficiently hydrophilic and could be miscible in any proportion with water.

**Figure 1:**
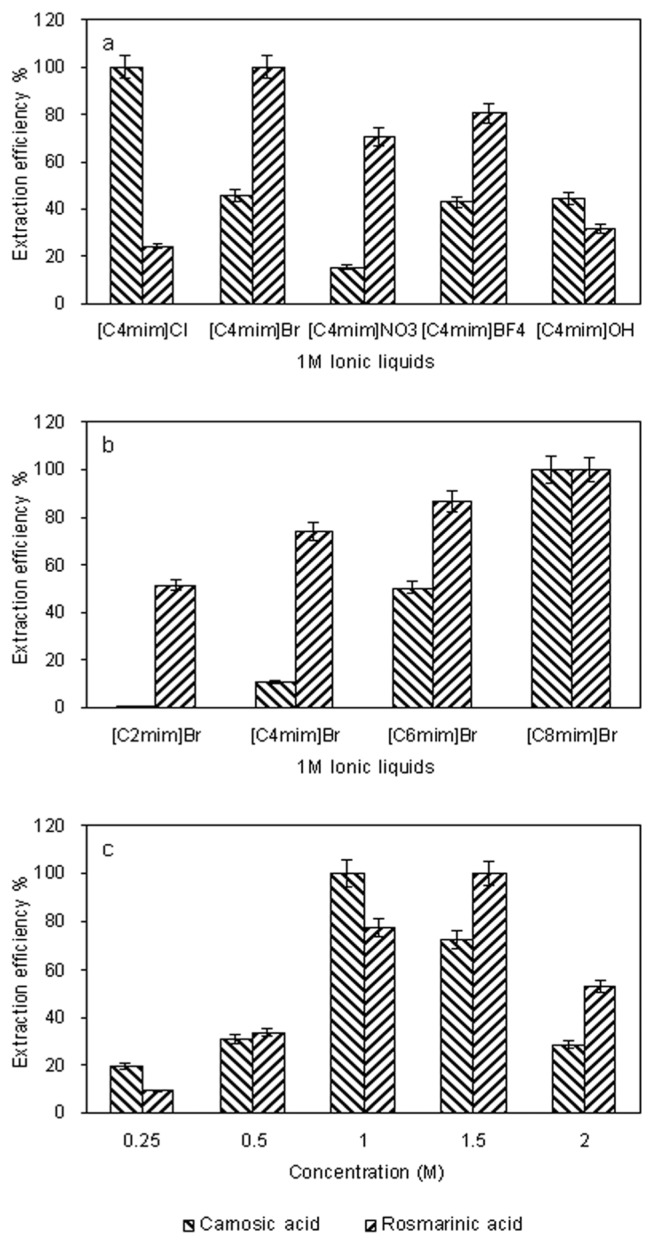
Effects of ionic liquids anion (**a**) the alkyl chain length of cation; (**b**) and ionic liquid concentration; (**c**) on the extraction efficiency of target compounds. The extraction efficiency is expressed as the observed values of target analytes and the maximum amount in each curve was taken to be 100%.

The results obtained are given in Figure 1a. The ionic liquids based on Cl^−^ and Br ^−^ were the most efficient of the liquids tested for carnosic acid; BF_4_^−^ and Br^−^ were the most efficient for rosmarinic acid, with Br^−^ showing the best results. This result indicates that extraction efficiency of phenolcarboxylic acids is anion-dependent, which is consistent with a previous study [25].

With the same anion of Br^−^, a series of *N*-methylimidazolium cations including 1-ethyl-3-methylimidazolium (C_2_mim^+^), 1-butyl-3-methylimidazolium (C_4_mim^+^), 1-hexyl-3-methylimidazolium (C_6_mim^+^), and 1-octyl-3-methylimidazolium (C_8_mim^+^) were used to investigate the effects of the alkyl chain length on the UAE of target compounds. As we could see in Figure 1b, the results indicated that the increasing alkyl chain length has significant influence on the extraction efficiency. This phenomenon could be attributed to the fact that increasing the alkyl chain length from ethyl to octyl decreases the water miscibility of the ionic liquids.

The excessive ionic liquid increases the viscosity of solution and decreases the penetration of ionic liquid into solid sample. Results shown in Figure 1c, indicated that for carnosic acid, the extraction efficiency increased when the [C_8_mim]Br concentration over the range of 0.25 M–2.0 M. When it further increased, however, slight decrease was observed. As for rosmarinic acid, the extraction efficiency could reach the maximum at the concentration of 1.5 M. The average extraction efficiency was almost the same at the concentrations of 1.0 M and 1.5 M. In order to save costs, 1.0 M of [C_8_mim]Br was selected. Considering the effect of both anion and cation, 1.0 M [C_8_mim]Br was selected for the subsequent experiments.

### 2.2. Optimization of Extraction Conditions

The univariate method is used in all instances for optimization of the five parameters, which might extensively affect the extraction step: solid–liquid ratio, soaking time, ultrasound power and ultrasound time.

There are many factors affecting the extraction of carnosic acid and rosmarinic acid from *R. officinalis*. We work out the optimum levels of each factor by the single factor test. However, because of the interaction among the factors, the combination impacts of the optimum levels of each factor may not be the optimum extraction conditions. To further study the interaction between the factors, we optimized the operating conditions by RSM and use the Box-Behnken software in data processing. Box-Behnken design with three factors was applied using Design-Expert 7.0 without any blocking. The bounds of the factors were 20–40 min ultrasound time, 1:15–1:25 ratio of solid–liquid, and 150–250 W ultrasound power.

#### 2.2.1. Single Factor Experiments

**Figure 2:**
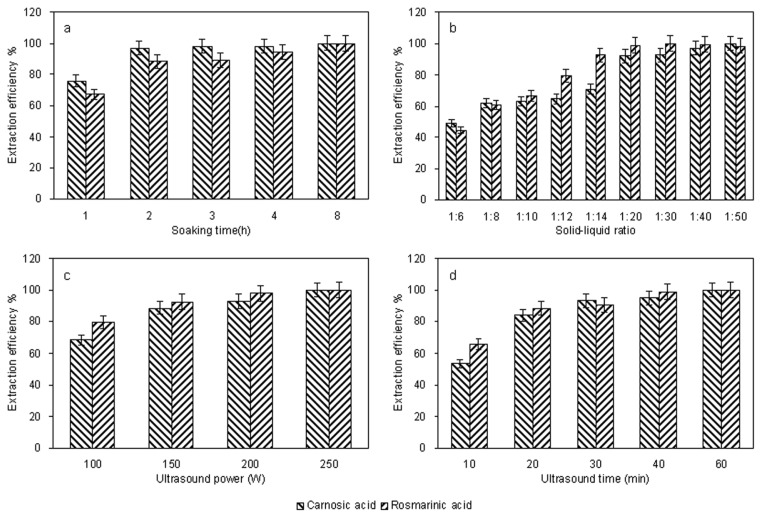
Effect of soaking time (**a**) solid–liquid ratio; (**b**) ultrasound power; (**c**) and ultrasound time; (**d**) on the extraction efficiency of target compounds with 1.0 M [C_8_mim]Br. The extraction efficiency is expressed as the observed values of target compounds and the maximum amount in each curve was taken to be 100%.

Experiments were conducted by soaking the dry herb powder in the ionic liquid solution for 1, 2, 3, 4, 8 h before UAE. Figure 2a shows the effect of soaking the herb in 1.0 M [C8mim]Br on the extraction of carnosic acid and rosmarinic acid from *R. officinalis* at room temperature (approx. 25 °C). It demonstrates the substantial increase in extraction efficiency obtained after soaking the herb. To extract phenolcarboxylic acids from the cellular structure, the solvent must have access to the cellular compartments where the phenolcarboxylic acids are located. An intact cell structure restricts accessibility of the solvent to the phenolcarboxylic acids, while ultrasound treated cells have a more open, fragmented structure, which facilitates efficient extraction. The increase in extraction efficiency of the phenolcarboxylic acids after soaking with the solvent is probably because of increased diffusion of the solvent into the cellular structure allowing improved solubilization of the phenolcarboxylic acids. The phenolcarboxylic acid extraction efficiency increased significantly when the soaking time was 0–2 h, however longer soaking times did not lead to further increases in efficiency. Hence 2 h was chosen as the optimal soaking time.

The solid–liquid ratio is also an important factor in the extraction. In general, a higher solvent volume can dissolve target compounds more effectively and result in a better extraction yield. A series of extractions were carried out with different solid–liquid ratios (1:6, 1:8, 1:10, 1:12, 1:14, 1:20, 1:30, 1:40, and 1:50 g/mL) to evaluate the effect of the solid–liquid ratio. Data shown in Figure 2b indicated an obvious increase of extraction efficiency of the target compounds before the solid–liquid ratio reached 1:20. When the solid-liquid ratio was increased from 1:20 to 1:50, however, the efficiency was not significantly improved. Thus, a solid-liquid ratio range of 1:15–1:25 is used in the further optimization study.

To examine the effect of the ultrasound power on the extraction efficiency, the effect of ultrasound power on UAE was explored with solid-liquid ratio of 1:20 and other conditions fixed as mentioned previously (solvent: 1.0 M [C_8_mim]Br, soaking time: 2 h, ultrasound time: 30 min). Extractions were carried out at 100, 150, 200 and 250 W, respectively. The results were shown in Figure 2c. From the microcosmic point of view, with the increase of ultrasound power, destruction of cell walls was more serious by the ultrasound energy [31,32]. The higher the ultrasound power was, the more solvent could enter cells and the more target compounds could permeate cell membranes, which suggested that increasing ultrasound power could enhance compounds yield. 150–250 W is taken for further optimization experiments.

Traditionally, higher extraction efficiency requires a longer extraction period. To investigate the influence of ultrasound time on extraction efficiency of phenolcarboxylic acids, 0.5 g sample was extracted at the conditions of 10 mL of 1.0 M [C_8_mim]Br, soaking 2 h, 250 W at different time (10, 20, 30, 40 and 60 min). The results shown in Figure 2d clearly indicated that when ultrasound time increased from 10 to 30 min, the extraction efficiency of the two target compounds increased dramatically. When the ultrasound time was longer than 30 min, the time effect was negligible. In view of this, a 20–40 min treatment time was selected for further optimization experiments.

#### 2.2.2. Optimization Parameters by Response Surface Methodology

To further study the interactions between the factors, we optimized the ultrasound time, solid–liquid ratio and ultrasound power by RSM. In Table 1, the maximum value of extraction efficiency was defined as 100%, and compared with the other values. From Table 2, the Model *F*-value of 389.49 indicated that the model was significant; there was only a 0.01% chance that a “Model *F*-Value” this large could occur due to statistical noise. Values of “Probability > *F*” less than 0.050 indicated model terms were significant. In this case, *X*_1_, *X*_2_, *X*_3_, *X*_1_*X*_2_, *X*_1_*X*_3_, *X*_1_^2^, *X*_2_^2^ and *X*_3_^2^ were significant model terms. The values that greater than 0.100 indicated that the model terms were not significant. If there were many insignificant model terms (not counting those required to support hierarchy), a model reduction (or refinement) was necessary. The “Lack of Fit *F*-value” of 1.61 implied the Lack of Fit was not significant relative to the pure error. There is a 32.07% chance that a “Lack of Fit *F*-value” this large could occur due to noise. Non-significant lack of fit is good-we want the model to fit. The “Pred *R*^2^” of 0.981 was in reasonable agreement with the “Adj *R*^2^” of 0.995. “Adeq Precision” measured the signal to noise ratio. A ratio greater than 4 was desirable. The present ratio of 50.947 indicated an adequate signal. This model can be used to navigate the design space.

The final average extraction efficiency of carnosic acid and rosmarinic acid (*Y*) was given by

(1) $$Y=99.08-1.43X_{1}-1.17\;X_{2}+5.64\;X_{3}-1.76X_{1}X_{2}+3.88\;X_{1}X_{3}+0.46\;X_{2}X_{3}-13.82{X_{1}}^{2}-15.81\;{X_{2}}^{2}-7.30\;{X_{3}}^{2}$$

**Figure 3:**
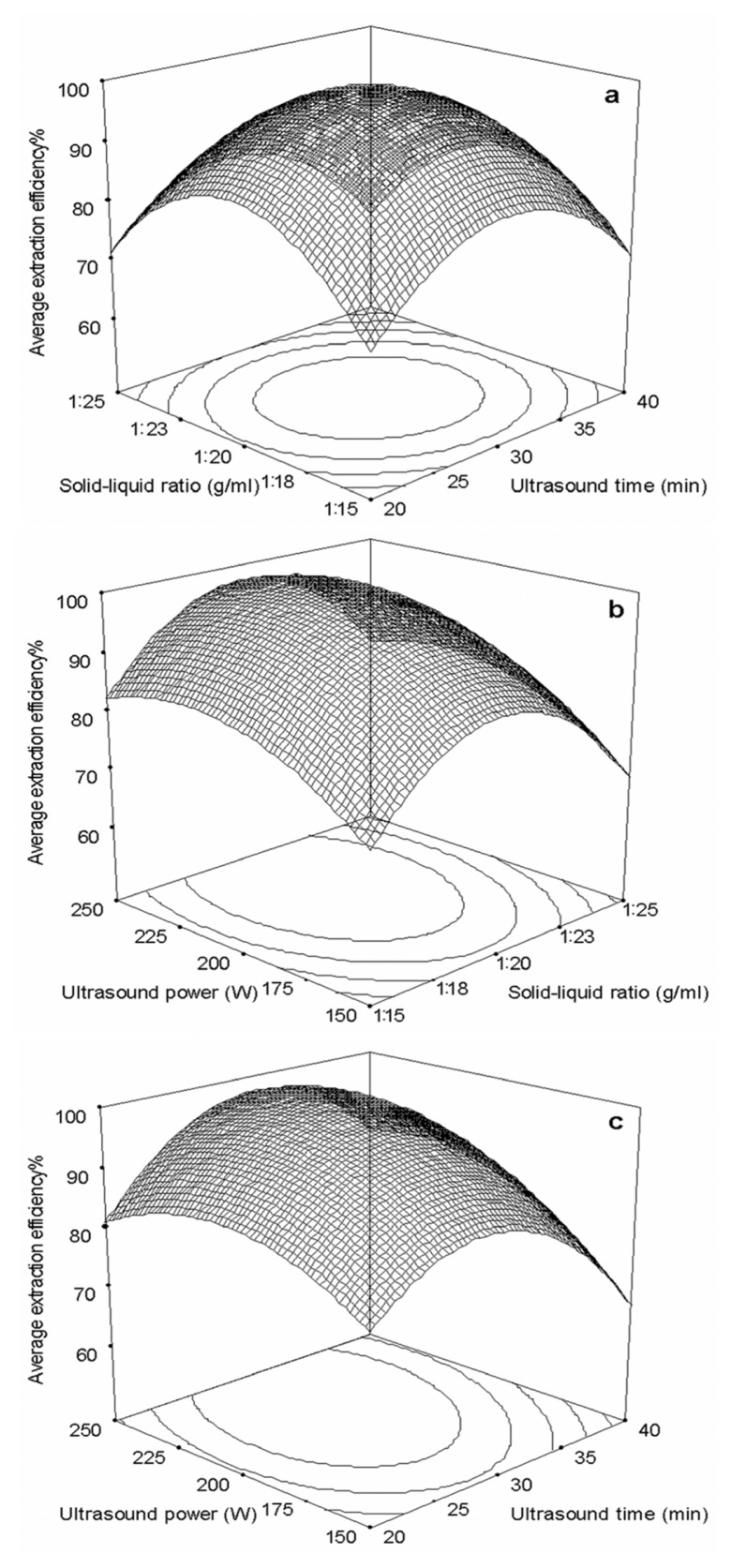
Response surface plots showing the effects of variables on average extraction efficiency of target compounds. (**a**) Interaction of solid-liquid ratio and ultrasound time; (**b**) Interaction of ultrasound power and solid-liquid ratio; (**c**) Interaction of ultrasound power and time. The average extraction efficiency is expressed as the observed values of target compounds and the maximum amount in each curve was taken to be 100%.

The response surfaces for the effect of independent variables on average extraction efficiency of carnosic acid and rosmarinic acid were showed in Figure 3. Figure 3a–c presented the interaction of ultrasound time and solid–liquid ratio, ultrasound power and solid–liquid ratio, ultrasound power and ultrasound time, respectively.

The conditions for point prediction by software were: 30 min ultrasound time at 221 W and 1:20 solid–liquid ratio. Under the conditions of point prediction, the extraction efficiency can reach to 100.17%.

### 2.3. Verification Tests

The verification tests were operated three times under the conditions of point prediction by RSM (30 min ultrasound time at 220 W and 1:20 solid–liquid ratio). The actual extraction efficiency was 98.91% with an error about 1.3%.

### 2.4. Comparison of ILUAE Approach with the Reference and Conventional Methods

In order to further demonstrate the use of ionic liquids, the proposed approach was then compared with the reference methods. All experiments were carried out under the same UAE conditions except the extractant, which included pure water, 1.0 M sodium chloride and 80% ethanol. Pure water is the most common and cheap solvent, so often selected as the cooperation, in all sorts of solvent extraction process. The main technical parameters used are listed in Table 3. The results shown in Table 3 indicated that, the extraction efficiency of carnosic acid and rosmarinic acid were only 0 and 61.03% ± 3.45% with pure water respectively, while that obtained when using 1.0 M [C8mim]Br were 66.23% ± 3.85% and 100.00% ± 4.76%. The solvent effect of the ionic liquid was therefore more important in achieving high extraction efficiency than the water in the ionic liquid–water system. While the two target compounds extraction efficiency achieved using 1.0 M sodium chloride solution was only 0 and 53.54% ± 3.38% respectively. The solvent effect of the ionic liquid was therefore more important in achieving high extraction efficiency than the salt effect derived from sodium chloride. Hence, salt effects do not play a major role in improving the extraction of carnosic acid and rosmarinic acid from *R. officinalis*.

The selection of an extraction method would mainly depend on the advantages and disadvantages of the processes, such as extraction efficiency, complexity, production cost, environmental friendliness and safety. For the comparison of the extraction efficiency of ILUAE with other five different conventional extraction techniques using optimal conditions were carried out to extract the two target compounds from *R. officinalis*. The results shown in Table 3 indicated that the average extraction efficiency of the two target compounds obtained using ILUAE and 80% ethanol UAE methods were higher than those achieved using 80% ethanol HRE, 80% ethanol ME, 80% ethanol SE and 80% ethanol SLE methods. Due to mechanical effects on cell walls evidenced by scanning electron microscopy [41,42], UAE can save a lot of time and solvent as compared to HRE, ME, SE and SLE method and bring higher extraction efficiency than HRE, ME, SE and SLE method. When the flammability of ethanol is considered, it is clear that ILUAE represents an environmentally friendly and efficient method for the extraction of carnosic acid and rosmarinic acid from *R. officinalis*.

### 2.5. Method Validation

**Figure 4:**
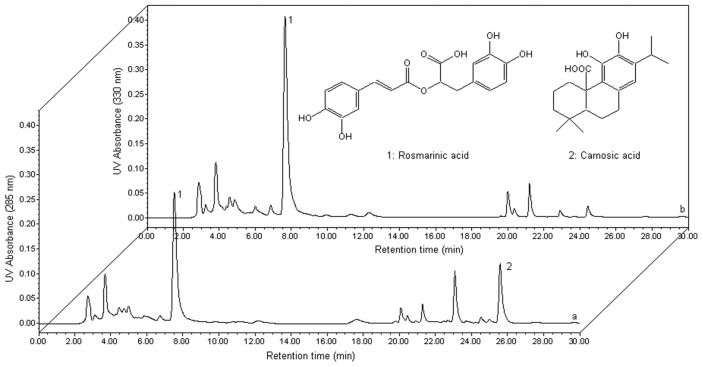
High-performance liquid chromatography (HPLC) profile of target compounds in an extract obtained using 1.0 M [C_8_mim]Br as extraction solvent. Inset: chemical structures of the target compounds.

To evaluate the proposed ILUAE approach, some parameters such as linearity, reproducibility, stability, and recovery were determined under the above optimized conditions. Calibration curves were obtained by dissolving the standard of carnosic acid to mobile phase at seven concentrations in the range of 0.1–20.0 mg/mL under the same HPLC conditions for *R. officinalis* extraction. Linear regression equation and correlation coefficients is *Y_ca_* = 2657149.4*X* − 202756.2 and 0.999, respectively. The limit of detection (LOD) and the limit of quantification (LOQ) were 6.23 μg/mL and 22.65 μg/mL, respectively. The linearity plotting was *Y_ra_* = 20470465.5*X* + 1625134.1 (*R*^2^ = 0.998) over the concentration range from 0.01 to 2.00 mg/mL, where *X* was rosmarinic acid concentration as mg/mL and *Y_ra_* was the peak area. The limit of detection was 0.81 μg/mL which was evaluated on the basis of a signal-to-noise ratio of 3, and the limit of quantification was 2.59 μg/mL. The reproducibility study was carried out on three repeated extraction with all methods. A comparison of the chromatograms of the two phenolcarboxylic acids obtained from standard solutions with that contained in the 1.0 M [C_8_mim]Br extract is shown in Figure 4.

As can be seen in Table 4, the relative standard deviation (RSD) obtained by the proposed approach is 0.78% and 2.49%. The results indicated that the repeatability of the method was good. Under the optimized conditions detailed above, the extracts were spiked with known quantities of standards as shown in Table 5. The recoveries of the carnosic acid and rosmarinic acid were in the range of 101.3% and 99.3%.

Previous articles claimed that ultrasound may cause degradations [43,44]. For this reason, the stability of the two target compounds under the experimentally derived optimum conditions was assessed by subjecting standards of carnosic acid and rosmarinic acid to UAE for 30 min at an ultrasound power of 220 W. The recovery of carnosic acid and rosmarinic acid was assumed to be indicative of the stability of them under the extraction conditions used (Table 5). The average complete recovery under the operating extraction conditions were 99.86% for carnosic acid and 99.22% for rosmarinic acid (Table 4) with no change in retention time observed for the two target compounds. Therefore degradation is insignificant under the selected optimum conditions. The standards in the 1.0 M [C_8_mim]Br solution stored for 15 days, the average recovery of carnosic acid and rosmarinic acid was 88.17% and 93.93%, respectively. The method validation studies indicated that the proposed method was credible.

## 3. Experimental Section

### 3.1. Materials and Reagents

Fresh leaves of the cultivated *R. officinalis* were hand-harvested in September from the Botanical Garden of the Zhejiang Hisun Pharmaceutical Co., Ltd., Hangzhou, China. All samples were air dried, milled, passed through 60 mesh screen and stored in closed desiccators before using. The same batch of sample was used here in the experiments, and the moisture content of air dried sample is 8.73%.

Reference compounds of carnosic acid and rosmarinic acid were purchased from Sigma-Aldrich Inc. (St. Louis, MO, USA). Acetonitrile and formic acid of HPLC grade were purchased from J & K Chemical Ltd. (Beijing, China). All ionic liquids ([C_4_mim]Cl, [C_2_mim]Br, [C_4_mim]Br, [C_6_mim]Br, [C_8_mim]Br, [C_4_mim]BF_4_, [C_4_mim]OH, [C_4_mim]NO_3_, where C_2_mim = 1-ethyl-3-methylimidazolium, C_4_mim = 1-butyl-3-methylimidazolium, C_6_mim = 1-hexyl-3-methylimidazolium, C_8_mim = 1-octyl-3-methylimidazolium) were bought from Chengjie (Shanghai, China). All other reagents were analytical grade and purchased from Tianjin Chemical Reagents Co. (Tianjin, China). Deionized water was purified by a Milli-Q water purification system (Millipore, MA, USA). All solutions and samples prepared for HPLC were filtered through 0.45 μm nylon membranes (Millipore, MA, USA) before used.

### 3.2. Apparatus

The extraction procedure was carried out in an ultrasound washing equipment (KQ-250DB, Kunshan Ultrasound Equipment Co., Ltd., Jiangsu, China). The bath was a rectangular container (23.5 × 13.3 × 10.2 cm), where 50 kHz transducers were annealed at the bottom. The bath power rating was 250 W on the scale of 40%–100%. The temperature of which was controlled by the alternative between inlet and outlet water. The high-performance liquid chromatography (HPLC) system (Waters, USA) was equipped with a 1525 binary HPLC pump, a 717 plus autosampler, a 717 automatic column temperature control box and 2487 dual λ absorbance detector. Chromatographic separation was performed on an Aichrom Bond-AQ C_18_ reversed-phase column (4.6 × 250 mm, 5 μm, Eka Nobel, Sweden).

### 3.3. HPLC Analysis and Quantification

The mobile phase used was composed of acetonitrile (A) and 2% formic acid aqueous solution (B) using the following gradient elution program for separation: 0–10 min, 30% (A); 10–15 min, 30%–80% (A); 15–25 min, 80% (A); 25–30 min, 80%–30% (A). The flow rate used for chromatography was 1 mL/min, and the injection volume was 10 μL. The UV detection wavelengths were 285 nm for carnosic acid and 330 nm for rosmarinic acid, the column temperature was ambient and the running time was 30 min. For standard sample solution, various amounts of carnosic acid and rosmarinic acid were dissolved in methanol to yield the stock solutions at concentration of 20 and 2 mg/mL, respectively. The retention times of carnosic acid and rosmarinic acid were 25.8 and 7.7 min, respectively. Under these conditions, the two phenolcarboxylic acids were resolved sufficiently to give baseline separation. Carnosic acid and rosmarinic acid were identified by comparing their retention times with standard solutions.

### 3.4. Ionic Liquid-Based Ultrasound-Assisted Extraction (ILUAE)

0.5 g of sample powder was soaked in 10 mL of the various ionic liquid aqueous solutions in a 25 mL flask. The flask was then partially suspended in the ultrasound bath, which contained 2500 mL of water. The suspension was then extracted by UAE, with temperature control achieved by the replacement of inlet and outlet water to avoid water temperature rises. The anion, cation, ionic liquid concentration, ultrasound power and time, soaking time and solid–liquid ratio were optimized to obtain the best extraction efficiency. After each extraction, the extract was filtered through a nylon membrane and then analyzed by HPLC.

Extraction process (a, b) were performed in an ultrasound unit with a power of 250 W, 0.5 g of dried sample was mixed with 10.0 mL ionic liquid in water at 1.0 M and then soaked for 2.0 h, before the suspension was extracted for 30 min by UAE. Extraction process (c) was performed in an ultrasound unit with a power of 250 W. 0.5 g of dried sample was mixed with 10.0 mL ionic liquid in water at different concentrations (0.25, 0.5, 1.0, 1.5 and 2.0 M) and then soaked for 2.0 h, before the suspension was extracted for 30 min by UAE (Figure 1).

### 3.5. Optimization of ILUAE by Response Surface Methodology

RSM was used to determine the optimum conditions for extraction of carnosic acid and rosmarinic acid from *R. officinalis.* The experimental design and statistical analysis were done using Stat-Ease software (Design-Expert 7.0, Delaware, USA). A three-level three-factor Box–Behnken design was chosen to evaluate the combined effect of three independent variables, ultrasound time, solid–liquid ratio, and ultrasound power. There were termed as *X*_1_, *X*_2_ and *X*_3_, respectively. The complete design consisted of 17 combinations, including five replicates of the center point (Table 1), and the response function (*Y*) was partitioned into linear, quadratic, and interactive components:

(2) $$Y=\beta_{0}+\sum_{i=1}^{k}B_{i}X_{i}+\sum_{i=1}^{k}B_{ii}X_{i}^{2}+\sum_{i>j}^{k}B_{ij}X_{i}X_{j}$$

where *Y* stands for average extraction efficiency of carnosic acid and rosmarinic acid; *β*_0_ denotes the model intercept; *B_i_*, *B_ii_*, and *B_ij_* represent the coefficients of the linear, quadratic, and interactive effects, respectively; *X_i_*, and *X_j_* are the coded independent variables; and *k* equals the number of tested factors (*k* = 3). Tables of the analysis of variance (ANOVA) were generated, and the effect and regression coefficients of individual linear, quadratic, and interactive terms were determined. The significances of all terms in the polynomial were evaluated statistically by computing the *F*-value at the probability (*p*) of 0.001, 0.01 or 0.05. The regression coefficients were then used to make statistical calculations to generate contour maps from the regression models.

### 3.6. Method Validation

Stability derived from test is performed by carnosic acid and rosmarinic acid standards dissolved in 1.0 M [C_8_mim]Br by UAE at the optimum conditions (2 h soaking time, 1:20 solid–liquid ratio, 30 min ultrasound time at 220 W).

The recoveries of carnosic acid and rosmarinic acid were taken as the indicative markers for the stability of carnosic acid and rosmarinic acid at the derived operating extraction conditions.

To determine the repeatability of the novel extraction method, six samples of the same weight (0.5 g) were processed under same optimum extraction conditions as those obtained from the systematic study of different extraction parameters.

### 3.7. Reference and Conventional Extraction Methods

Pure water, 1.0 M sodium bromide and 80% ethanol were selected for use as reference solvents in the UAE of carnosic acid and rosmarinic acid from *R. officinalis*. The extraction experiments were operated under the optimized conditions except for solvent type. The process of extraction: 0.5 g sample powder was mixed with 10 mL of the above solvents and soaked for 2 h in 25 mL flask. The suspension was then extracted for 30 min by ultrasound power of 250 W. All the final extracts above were also filtrated through a 0.45 μm filter for subsequent HPLC analysis. Aqueous ethanol (80%) was selected as solvents for HRE, ME, SE and SLE.

### 3.8. Statistical Analysis

The one way ANOVA test was used to calculate the significance of the differences of extraction efficiency. The results of HPLC analysis were expressed as means of extraction efficiency ± SD.

## 4. Conclusions

An efficient method has been developed for the extraction of carnosic acid and rosmarinic acid from *R. officinalis*. The optimum conditions for ILUAE were studied. Under the optimized conditions, satisfactory extraction efficiency of the two phenolcarboxylic acids was obtained. Relative to other methods, the proposed approach provides higher extraction efficiency and significantly reduced extraction time. The extraction of carnosic acid and rosmarinic acid can therefore be readily and efficiently achieved by ILUAE using the method developed in this study. The method may also prove useful in the development of energy saving and environmentally friendly extraction methods for other phenolcarboxylic acids.

## Figures and Tables

**Table 1:** Experimental data and the observed response value with different combinations of ultrasound time (*X*_1_), solid–liquid ratio (*X*_2_) and ultrasound power (*X*_3_) used in the Box–Behnken design.

Run	Independent variables			Response
	
	*X*_1_ (min)	*X*_2_ (g/mL)	*X*_3_ (W)	Average extraction efficiency (%) a
1	30 (0)	1:20 (0)	200 (0)	98.75
2	40 (1)	1:20 (0)	250 (1)	86.00
3	40 (1)	1:25 (1)	200 (0)	65.00
4	20 (−1)	1:25 (1)	200 (0)	72.10
5	30 (0)	1:20 (0)	200 (0)	99.30
6	30 (0)	1:25 (1)	150 (−1)	68.04
7	40 (1)	1:20 (0)	150 (−1)	67.77
8	20 (−1)	1:20 (0)	250 (1)	80.40
9	30 (0)	1:20 (0)	200 (0)	97.94
10	30 (0)	1:20 (0)	200 (0)	99.40
11	30 (0)	1:20 (0)	200 (0)	100.00
12	30 (0)	1:15 (−1)	250 (1)	82.99
13	40 (1)	1:15 (−1)	200 (0)	70.31
14	20 (−1)	1:20 (0)	150 (−1)	77.68
15	30 (0)	1:25 (1)	250 (1)	81.04
16	20 (−1)	1:15 (−1)	200 (0)	70.37
17	30 (0)	1:15 (−1)	150 (−1)	71.83

a The average extraction efficiency is expressed as the observed values and the maximum amount was taken to be 100%.

**Table 2:** Test of significance for regression coefficient a.

Source	Sum of squares	Degree of freedom	Mean square	*F*-value	*p*-value
Model b	2652.90	9	294.77	389.49	<0.0001
*X*_1_	16.41	1	16.41	21.69	0.0023
*X*_2_	10.86	1	10.86	14.36	0.0068
*X*_3_	254.26	1	254.26	335.97	<0.0001
*X*_1_*X*_2_	12.42	1	12.42	16.41	0.0049
*X*_1_*X*_3_	60.24	1	60.24	79.60	<0.0001
*X*_2_*X*_3_	0.85	1	0.85	1.12	0.3249
*X*_1_^2^	804.65	1	804.65	1063.23	<0.0001
*X*_2_^2^	1052.30	1	1052.30	1390.46	<0.0001
*X*_3_^2^	224.08	1	224.08	296.09	<0.0001
Residual	5.30	7	0.76		
Lack of Fit	2.90	3	0.97	1.61	0.3207
Pure Error	2.40	4	0.60		
Cor Total	2658.19	16			

Pred *R*^2^	0.981				
Adj *R*^2^	0.995				
Adeq Precision	50.947				

a The results were obtained with the Design Expert 7.0 software;

b *X*_1_ is ultrasound time (min), *X*_2_ is solid–liquid ratio (g/mL), *X*_3_ is ultrasound power (W).

**Table 3:** Comparison of ionic liquid-based ultrasound-assisted extraction (ILUAE) with other extraction methods, mean ± S.D. (*n* = 3).

No.	Solvent	Methods	Extraction time (h)	Solvent consumption (mL/g)	Extraction efficiency ± SD (%) a		

					Carnosic acid	Rosmarinic acid	Average
1	Pure water	UAE	0.5	20	0	61.03 ± 3.45	61.03 ± 3.45
2	1 M NaBr	UAE	0.5	20	0	53.54 ± 3.38	53.54 ± 3.38
3	80% Ethanol	UAE	0.5	20	100.00 ± 5.44	64.69 ± 4.23	82.35 ± 4.84
4	1 M [C_8_mim]Br	UAE	0.5	20	66.23 ± 3.85	100.00 ± 4.76	83.12 ± 4.31
5	80% Ethanol	HRE	4	20	99.05 ± 4.87	67.61 ± 3.42	74.87 ± 4.15
6	80% Ethanol	ME	48	20	95.04 ± 4.33	40.08 ± 2.66	48.69 ± 2.50
7	80% Ethanol	SE	24	20	96.80 ± 3.96	67.03 ± 3.78	65.96 ± 3.87
8	80% Ethanol	SLE	24	20	95.47 ± 3.34	62.22 ± 4.05	70.35 ± 3.70

a The extraction efficiency is expressed as the observed values of target compounds and the maximum amount in each curve was taken to be 100%.

**Table 4:** Stability studies of standards carnosic acid and rosmarinic acid under optimum ultrasound-assisted extraction (UAE) conditions. Extraction conditions: 250 W ultrasound power, 30 min ultrasound time, 10 mL solution volume prepared with 1.0 M [C_8_mim]Br.

Compounds	Initial concentration (mg/mL)	Recovered concentration after UAE (mg/mL)	RSD% (*n* = 3)	Average recovery (%)	Recovered concentration after 3 day (mg/mL)	RSD% (*n* = 3)	Average recovery (%)	Recovered concentration after 15 day (mg/mL)	RSD% (*n* = 3)	Average recovery (%)
Carnosic acid	14.16	14.14	3.21	99.86	13.66	1.06	96.48	12.48	2.11	88.17
Rosmarinic acid	1.28	1.27	0.78	99.22	1.25	2.49	98.01	1.20	1.60	93.93

**Table 5:** The recovery of carnosic acid and rosmarinic acid from dried leaves of *R. officinalis* (*n* = 3).

Sample	Phenolcarboxylic acids content of the sample determined (mg)		The amount of added phenolcarboxylic acids standards (mg)		The amount of the sample determined with added phenolcarboxylic acids standards (mg)		Recovery (%)	

	Carnosic acid	Rosmarinic acid	Carnosic acid	Rosmarinic acid	Carnosic acid	Rosmarinic acid	Carnosic acid	Rosmarinic acid
1	7.8	12.0	5.0	10.0	13.3	21.5	103.9	97.7
2	7.8	12.0	10.0	15.0	17.2	26.6	96.6	98.5
3	7.8	12.0	15.0	20.0	23.6	33.5	103.5	101.6
Average	-	-	-	-	-	-	101.3	99.3

## References

[1] Zunin P., Leardi R., Bisio A., Boggia R., Romussi G. (2010). Oxidative stability of virgin olive oil enriched with carnosic acid. Food Res. Int.

[2] Wang H., Zu G., Yang L., Zu Y., Wang H., Zhang Z., Zhang Y., Zhang L., Wang H. (2011). Effects of heat and ultraviolet radiation on the stability of pine nut oil supplemented with carnosic acid. J. Agric. Food Chem.

[3] Wang H., Wang H., Yang L., Zu Y., Liu F., Liu T. (2011). Comparative effect of carnosic acid, BHT and α-tocopherol on the stability of squalene under heating and UV irradiation. Food Res. Int.

[4] Wang H., Liu F., Yang L., Zu Y., Wang H., Qu S., Zhang Y. (2011). Oxidative stability of fish oil supplemented with carnosic acid compared with synthetic antioxidants during long-term storage. Food Chem.

[5] Bernardes W.A., Lucarini R., Tozatti M.G., Souza M.G.M., Silva M.L.A., Filho A.A.S., Martins C.H.G., Crotti A.E.M., Pauletti P.M., Groppo M. (2010). Antimicrobial activity of *Rosmarinus officinalis* against oral pathogens: Relevance of carnosic acid and carnosol. Chem. Biodivers.

[6] Poeckel D., Greiner C., Verhoff M., Rau O., Tausch L., Hörnig C., Steinhilber D., Schubert-Zsilavecz M., Werz O. (2008). Carnosic acid and carnosol potently inhibit human 5-lipoxygenase and suppress pro-inflammatory responses of stimulated human polymorphonuclear leukocytes. Biochem. Pharm.

[7] Yesil-Celiktas O., Sevimli C., Bedir E., Vardar-Sukan F (2010). Inhibitory effects of rosemary extracts, carnosic acid and rosmarinic acid on the growth of various human cancer cell lines. Plant Food Hum. Nutr.

[8] Manoharan S., Selvan M., Selvan V., Silvan S., Baskaran N., Singh A.K., Kumar V.V. (2010). Carnosic acid: A potent chemopreventive agent against oral carcinogenesis. Chem. Biol. Interact.

[9] Satoh T., Kosaka K., Itoh K., Kobayashi A., Yamamoto M., Shimojo Y., Kitajima C., Cui J., Kamins J., Okamoto S. (2008). Carnosic acid, a catechol-type electrophilic compound, protects neurons both *in vitro* and *in vivo* through activation of the Keap1/Nrf2 pathway via *S-*alkylation of targeted cysteines on Keap1. J. Neurochem.

[10] Ninomiya K., Matsuda H., Shimoda H., Nishida N., Kasajima N., Yoshino T., Morikawa T., Yoshikawa M. (2004). Carnosic acid, a new class of lipid absorption inhibitor from sage. Bioorg. Med. Chem. Lett.

[11] Shanlou Q., Weihua L., Ryoko T., Miyako H., Keiko M., Fumio T., Yukio N., Masataka Y. (2005). Rosmarinic acid inhibits the formation of reactive oxygen and nitrogen species in RAW264.7 macrophages. Free Radic. Res.

[12] Sui X., Liu T., Ma C., Yang L., Zu Y., Zhang L., Wang H. (2012). Microwave irradiation to pretreat rosemary (*Rosmarinus officinalis* L.) for maintaining antioxidant content during storage and to extract essential oil simultaneously. Food Chem.

[13] Osakabe N., Takano H., Sanbongi C., Yasuda A., Yanagisawa R., Inoue K., Yoshikawa T. (2004). Anti-inflammatory and anti-allergic effect of rosmarinic acid (RA); inhibition of seasonal allergic rhinoconjunctivitis (SAR) and its mechanism. Biol. Factors.

[14] Furtado M.A., de Almeida L.C.F., Furtado R.A., Cunha W.R., Tavares D.C. (2008). Antimutagenicity of rosmarinic acid in Swiss mice evaluated by the micronucleus assay. Mutat. Res. Gen. Toxicol. Environ.

[15] Huang S.S., Zheng R.L. (2006). Rosmarinic acid inhibits angiogenesis and its mechanism of action *in vitro*. Cancer Lett.

[16] Kim D.S., Kim H.R., Woo E.R., Hong S.T., Chae H.J., Chae S.W. (2005). Inhibitory effects of rosmarinic acid on adriamycin-induced apoptosis in H9c2 cardiac muscle cells by inhibiting reactive oxygen species and the activations of c-Jun *N*-terminal kinase and extracellular signal-regulated kinase. Biochem. Pharm.

[17] Li G.S., Jiang W.L., Tian J.W., Qu G.W., Zhu H.B., Fu F.H. (2010). *In vitro* and *in vivo* antifibrotic effects of rosmarinic acid on experimental liver fibrosis. Phytomedicine.

[18] Debersac P., Vernevaut M.F., Amiot M.J., Suschetet M., Siess M.H. (2001). Effects of a water-soluble extract of rosemary and its purified component rosmarinic acid on xenobiotic-metabolizing enzymes in rat liver. Food Chem. Toxicol.

[19] Fallarini S., Miglio G., Paoletti T., Minassi A., Amoruso A., Bardelli C., Brunelleschi S., Lombardi G. (2009). Clovamide and rosmarinic acid induce neuroprotective effects *in vitro* models of neuronal death. Br. J. Pharm.

[20] Lee J., Jung E., Koh J., Kim Y.S., Park D. (2008). Effect of rosmarinic acid on atopic dermatitis. J. Dermatol.

[21] Psotova J., Svobodova A., Kolarova H., Walterova D. (2006). Photoprotective properties of *Prunella vulgaris* and rosmarinic acid on humankeratinocytes. J. Photochem. Photobiol. B Biol.

[22] Hamaguchi T., Ono K., Murase A., Yamada M. (2009). Phenolic compounds prevent Alzheimer’s pathology through different effects on the amyloid-β aggregation pathway. Am. J. Pathol.

[23] Ma C., Wang S., Yang L., Zu Y (2012). Ionic liquid-based ultrasonic-assisted extraction of camptothecin and 10-hydroxycamptothecin from samara of *Camptotheca acuminate*. Chem. Eng. Process. Process Intensif.

[24] Wang S., Yang L., Zu Y., Zhao C., Sun X., Zhang L., Zhang Z. (2011). Design and performance evaluation of ionic liquids-microwave based environmental-friendly extraction technique for camptothecin and 10-hydroxycamptothecin from samara of *Camptotheca acuminate*. Ind. Eng. Chem. Res.

[25] Liu T., Sui X., Zhang R., Yang L., Zu Y., Zhang L., Zhang Y., Zhang Z. (2011). Application of ionic liquids based microwave-assisted simultaneous extraction of carnosic acid, rosmarinic acid and essential oil from *Rosmarinus officinalis*. J. Chromatogr. A.

[26] Jiao Y., Zuo Y. (2009). Ultrasonic extraction and HPLC determination of anthraquinones, aloe-emodine, emodine, rheine, chrysophanol, and physcione, in Radix *Polygoni multiflori*. Phytochem. Anal.

[27] Zuo Y., Zhang L., Wu J., Fritz J.W., Medeiros S., Rego C. (2004). Ultrasonic extraction and capillary gas chromatography determination of nicotine in pharmaceutical formulations. Anal. Chim. Acta.

[28] Chemat F., Zill-e-Huma, Khan M.K. (2011). Applications of ultrasound in food technology: Processing, preservation and extraction. Ultrason. Sonochem.

[29] Rodrigues S., Pinto G.A.S. (2007). Ultrasound extraction of phenolic compounds from coconut (*Cocos nucifera*) shell powder. J. Food Eng.

[30] Pereiro A.B., Rodríguez A. (2010). An ionic liquid proposed as solvent in aromatic hydrocarbon separation by liquid extraction. AIChE J.

[31] Hernández-Fernández F.J., de los Ríos A.P., Gómez D., Rubio M., Víllora G. (2010). Selective extraction of organic compounds from transesterification reaction mixtures by using ionic liquids. AIChE J.

[32] Yang L., Wang H., Zu Y., Zhao C., Zhang L., Chen X., Zhang Z. (2011). Ultrasound-assisted extraction of the three terpenoid indole alkaloids vindoline, catharanthine and vinblastine using ionic liquid solution from *Catharanthus roseus*. Chem. Eng. J.

[33] Cao X., Ye X., Lu Y., Yu Y., Mo W. (2009). Ionic liquid-based ultrasonic-assisted extraction of piperine from white pepper. Anal. Chim. Acta.

[34] Du F.Y., Xiao X.H., Li G.K. (2007). Application of ionic liquids in the microwave-assisted extraction of *trans*-resveratrol from *Rhizma Polygoni Cuspidati*. J. Chromatogr. A.

[35] Yang L., Sun X., Yang F., Zhao C., Zhang L., Zu Y. (2012). Application of ionic liquids in the microwave-assisted extraction of proanthocyanidins from *Larix gmelini* bark. Int. J. Mol. Sci.

[36] Wu K.K., Zhang Q.L., Liu Q., Tang F., Long Y.M., Yao S.Z. (2009). Ionic liquid surfactant-mediated ultrasonic-assisted extraction coupled to HPLC: Application to analysis of tanshinones in *Salvia miltiorrhiza* Bunge. J. Sep. Sci.

[37] Ma C., Liu T., Yang L., Zu Y., Wang S., Zhang R. (2011). Study on ionic liquid-based ultrasonic-assisted extraction of biphenyl cyclooctene lignans from the fruit of *Schisandra chinensis* Baill. Anal. Chim. Acta.

[38] Ma C., Liu T., Yang L., Zu Y., Chen X., Zhang L., Zhang Y., Zhao C. (2011). Ionic liquid based microwave simultaneous extraction of essential oil and biphenyl cyclooctene lignans from *Schisandra chinensis* Baill fruits. J. Chromatogr. A.

[39] Yang L., Liu Y., Zu Y., Zhao C., Zhang L., Chen X., Zhang Z. (2011). Optimize the process of ionic liquid-based ultrasonic-assisted extraction of aesculin and aesculetin from *Cortex Fraxini* by response surface methodology. Chem. Eng. J.

[40] Manic M.S., Najdanovic-Visak V., da Ponte M.N., Visak Z.P. (2011). Extraction of free fatty acids from soybean oil using ionic liquids or poly(ethyleneglycol)s. AIChE J.

[41] Balachandran S., Kentish S.E., Mawson R., Ashokkumar M. (2006). Ultrasonic enhancement of the supercritical extraction from ginger. Ultrason. Sonochem.

[42] Li H., Pordesimo L.O., Weiss J. (2004). High-intensity ultrasound assisted extraction of oil from soybeans. Food Res. Int.

[43] Chemat F., Grondin I., Costes P., Moutoussamy L., Sing A.S.C., Smadja J. (2004). High power ultrasound effects on lipid oxidation of refined sunflower oil. Ultrason. Sonochem.

[44] Chemat F., Grondin I., Sing A.S.C., Smadja J. (2004). Deterioration of edible oils during food processing by ultrasound. Ultrason. Sonochem.

